# Novel amino-β-lactam derivatives as potent cholesterol absorption inhibitors

**DOI:** 10.1016/j.ejmech.2014.10.014

**Published:** 2014-11-24

**Authors:** Tonko Dražić, Krešimir Molčanov, Vinay Sachdev, Martina Malnar, Silva Hećimović, Jay V. Patankar, Sascha Obrowsky, Sanja Levak-Frank, Ivan Habuš, Dagmar Kratky

**Affiliations:** a Ruđer Bošković Institute, Bijenička c. 54, HR-10002 Zagreb, Croatia; b Institute of Molecular Biology and Biochemistry, Medical University of Graz, Graz, Austria

**Keywords:** β-lactam, Cholesterol absorption inhibitor, Hyperlipidemia, Cardiovascular heart disease, TBDMSCl, *tert*-butyldimethylsilyl chloride, CBS, Corey–Bakshi–Shibata catalyst, CHD, cardiovascular heart disease, TEAA, tetraethylammonium acetate, FCS, fetal calf serum, MTT, 3-(4,5-dimethylthiazol-2-yl)-2,5-diphenyltetrazolium bromide, NPC1L1, Niemann-Pick C1-like protein 1, THAP, 2,4,6-trihydroxyacetophenone, DAC, ammonium citrate dibasic

## Abstract

Two new *trans*-(3*R*,4*R*)-amino-β-lactam derivatives and their diastereoisomeric mixtures were synthesized as ezetimibe bioisosteres and tested in *in vitro* and *in vivo* experiments as novel β-lactam cholesterol absorption inhibitors. Both compounds exhibited low cytotoxicity in MDCKII, hNPC1L1/MDCKII, and HepG2 cell lines and potent inhibitory effect in hNPC1L1/MDCKII cells. In addition, these compounds markedly reduced cholesterol absorption in mice, resulting in reduced cholesterol concentrations in plasma, liver, and intestine. We determined the crystal structure of one amino-β-lactam derivative to establish unambiguously both the absolute and relative configuration at the new stereogenic centre C17, which was assigned to be *S*. The p*K*_a_ values for both compounds are 9.35, implying that the amino-β-lactam derivatives and their diastereoisomeric mixtures are in form of ammonium salt in blood and the intestine. The IC_50_ value for the diastereoisomeric mixture is 60 μM. *In vivo*, it efficiently inhibited cholesterol absorption comparable to ezetimibe.

## Highlights

• We synthesized two novel 3-amino-β-lactam derivatives.
• X-ray structure of one novel compound was determined.
• Both novel compounds are in the form of NH_4_^+^ salt in blood and intestine.
• Novel compounds efficiently inhibit cholesterol absorption *in vitro* and *in vivo*.

## Graphical abstract

**Figure:**
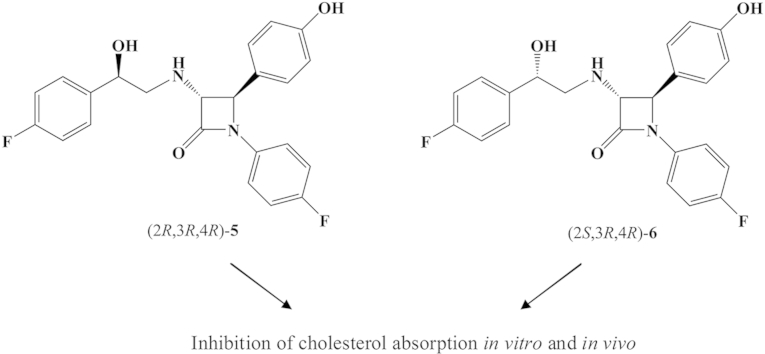

## 1 Introduction

**Fig. 1:**
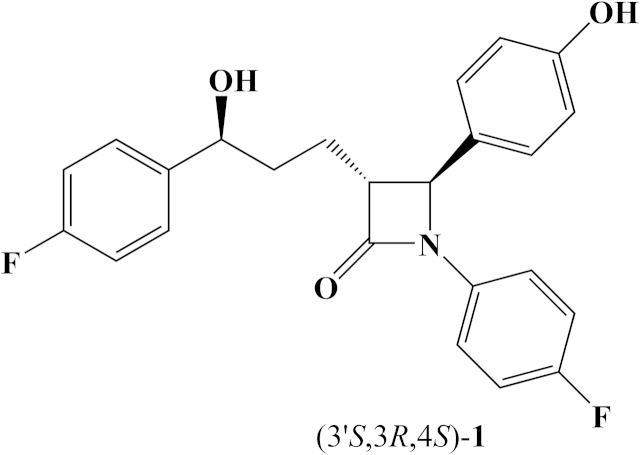
Structure of ezetimibe (3′*S*,3*R*,4*S*)-**1**.

Cardiovascular heart disease (CHD) is the leading cause of death worldwide [1]. One of the major risk factors for CHD are elevated serum cholesterol concentrations [2]. Lowering the level of serum cholesterol is an established clinical practice for CHD treatment, intervention, and prevention. Pharmacologically, circulating cholesterol concentrations are reduced by statins, which are 3-hydroxy-3-methylglutaryl coenzyme A (HMG-CoA) reductase inhibitors, affecting biosynthesis of endogenous cholesterol [3], [4]. Another approach is to block the absorption of dietary cholesterol, which is the other major contributor to serum cholesterol concentrations in the small intestine. Ezetimibe **1** (Zetia, Ezetrol; approved in 2002) (Fig. 1) is the only cholesterol absorption inhibitor on the market today [5]. It can be applied either as a monotherapy or in combination with statins [6]. Ezetimibe **1** was originally discovered without a known molecular target through *in vivo* screening of cholesterol-fed hamsters [7]. In 2004, researchers from the Schering-Plough Research Institute identified Niemann Pick C1-like1 (NPC1L1) protein as a molecular target of ezetimibe **1** [8]. Ezetimibe **1** acts by blocking the internalization of NPC1L1, thereby preventing cholesterol from entering the cytoplasm of enterocytes [9].

A thorough structure–activity relationship (SAR) study [5] of the β-lactam cholesterol absorption inhibitors in cholesterol-fed hamsters revealed that the 2-azetidinone backbone as well as the aryl group at the N-1 and C-4 position of the β-lactam ring are required for activity. The aryl group at the C-4 position of the β-lactam ring is optimally substituted with alkoxy or hydroxy groups at the *para*-position. The side chain at the C-3 position of the β-lactam ring with three linking atoms bearing a pendent aryl group is optimal. Preferred configuration at the C-4 chiral center of the β-lactam ring is *S* and the C-3 atom tolerates *S* or *R* configurations [5]. Introduction of a heteroatom at the 1′-position of the C-3 side chain can also contribute to the activity, whereas isosteric groups at the 3′-position of the side chain decrease the activity [10].

**Fig. 2:**
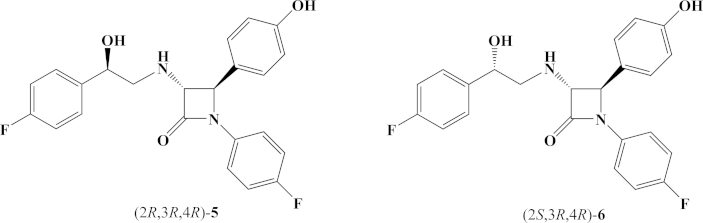
Structure of novel amino bioisosteres (2*R*,3*R*,4*R*)-**5** and (2*S*,3*R*,4*R*)-**6**.

In continuation of our research in the field of β-lactam chemistry [11], [12], [13], [14], [15] and taking into consideration the requirements determined by SAR studies [5], we synthesized bioisosteres **5** and **6** (Fig. 2) of ezetimibe **1** bearing a –NH– group at the C-3 position of the β-lactam ring.

Bioisosterism is a useful approach for lead compound modification that can result in improved pharmacological activity, decreased toxicity, and optimized pharmacokinetics. With the classical bioisosteric exchange of the –CH_2_– with a –NH– group we aimed at investigating whether the change in polarity of the side chain, the ability of additional H-bond, and ammonium salt formations would affect their cholesterol absorption inhibition and cytotoxicity. Here we show that our new ezetimibe analogs **5**, **6** and their diastereoisomeric mixture **5**/**6** (70:30) are potent novel cholesterol absorption inhibitors.

## 2 Results and discussion

### 2.1 Chemistry

#### 2.1.1 Synthesis

**Fig. 3:**
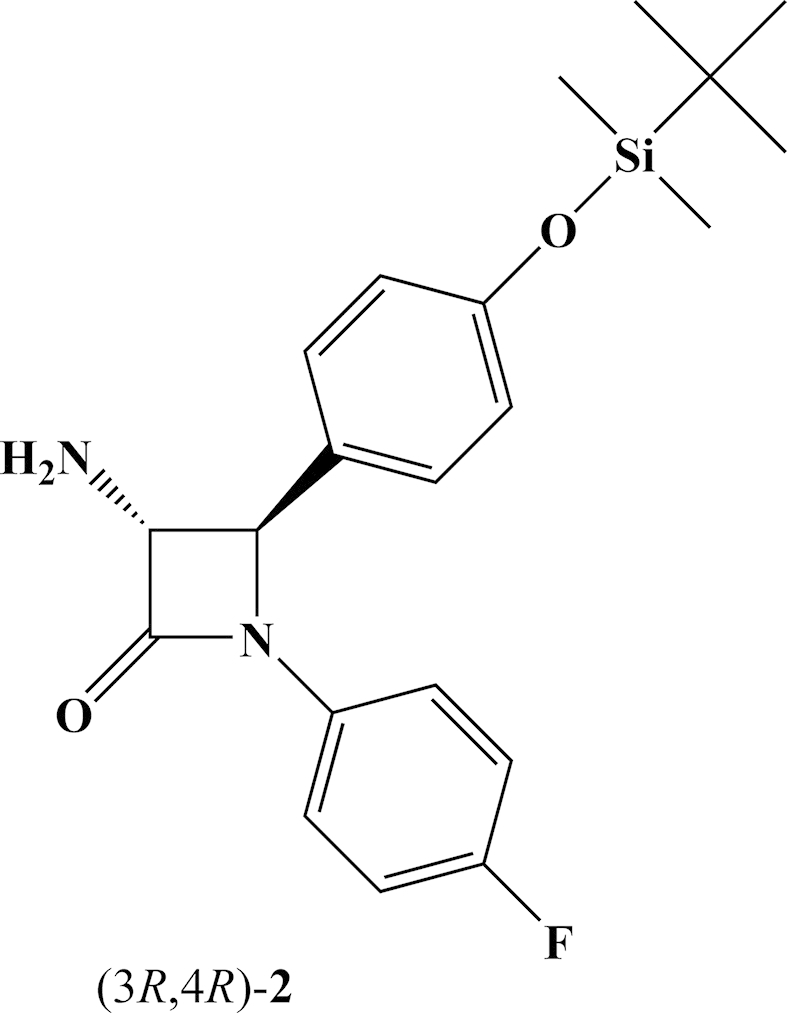
*trans*-(3*R*,4*R*)-amino-β-lactam **2**.

We synthesized two novel ezetimibe analogs **5** and **6** (Fig. 2) and their diastereoisomeric mixtures **5**/**6** (70:30) and **6**/**5** (85:15) from enantiomerically pure *trans*-(3*R*,4*R*)-amino-β-lactam **2** (Fig. 3) and determined their *in vitro* and *in vivo* activities as cholesterol absorption inhibitors. We extensively studied the stereoselectivity of the side chain keto group reduction with CBS-catalyst in proximity of the –NH– group at the C-3 position of 2-azetidinone. Enantiomerically pure *trans*-(3*R*,4*R*)-amino-β-lactam **2** (Fig. 3) was synthesized applying the chiral ester enolate-imine condensation [16], [17]. Enantiomeric purity of **2** (>99% ee) was determined by the Mosher's MTPA method [18] on ^19^F NMR (−67.79 ppm, s, 3F, CF_3_ and -116.22 ppm, s, 1F, N–C_6_H_4_F).

There are two possible approaches for the synthesis of ezetimibe analogs **5** and **6** and their diasteroisomeric mixtures **5**/**6** and **6**/**5** from *trans*-amino-β-lactam **2**: (**i**) *N*-alkylation of *trans*-amino-β-lactam **2** with commercially available 2-bromo-1-(4-fluorophenyl)ethan-1-one **3** followed by stereoselective reduction of the side chain keto group at the C-3 of the β-lactam ring with CBS-catalyst or (**ii**) stereoselective reduction of the keto group of **3** preceding *N*-alkylation reaction. In the present study we applied both approaches.

**Scheme 1:**
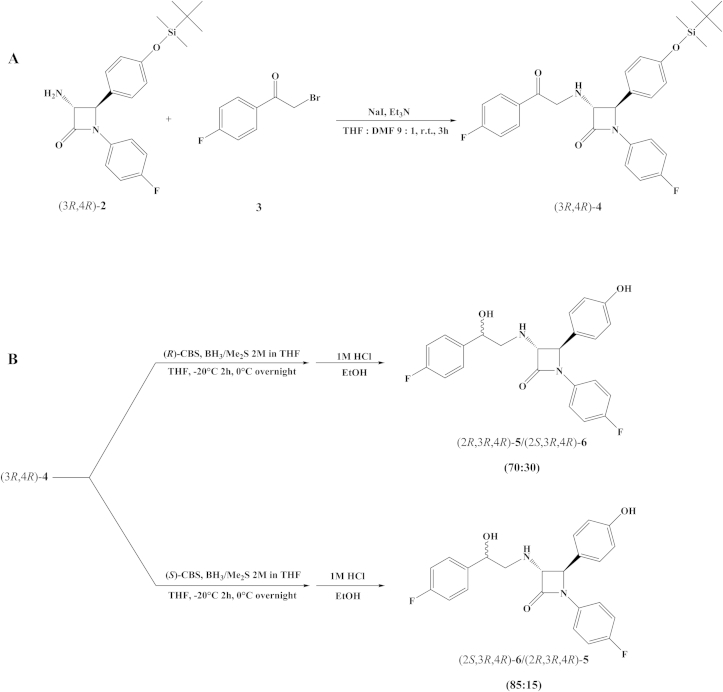
(**A**) *N*-alkylation of *trans*-amino-β-lactam (3*R*,4*R*)-**2** and (**B**) subsequent reduction of the keto group in (3*R*,4*R*)-**4** with CBS-catalysts.

**Fig. 4:**
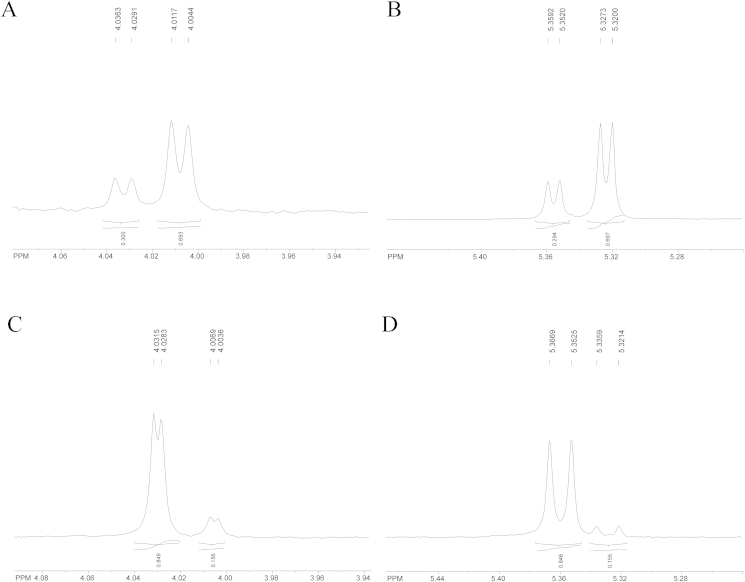
^1^H NMR spectra of the (**A**) C-4 β-lactam (300 MHz, CDCl_3_) and (**B**) side chain C-2 O*H* group (300 MHz, DMSO-*d*_*6*_) proton signals for 5/6 (70:30) and (**C**) C4 β-lactam (600 MHz, CDCl_3_) and (**D**) side chain C-2 O*H* group (300 MHz, DMSO-*d*_*6*_) well-resolved proton signals for **6**/**5** (85:15).
(ii) Stereoselective reduction of ketone 3 followed by *N*-alkylation.

(i) *N*-alkylation of *trans*-amino-β-lactam 2 with ketone 3 and subsequent reduction of the side chain keto group. *N*-alkylation of *trans*-amino-β-lactam **2** was performed in mild conditions using NaI for *in situ* generation of 2-iodo-derivative of 2-bromo-1-(4-fluorophenyl)ethan-1-one **3** in the presence of Et_3_N at room temperature and provided **4** in a moderate yield (46%) (Scheme 1A). A mixture of THF and DMF in ratio 9:1 was found optimal for the reaction. The C-3 side chain hydroxy group was obtained by stereoselective reduction of the keto group with CBS catalyst (Scheme 1B) [19], [20], [21]. However, addition of CBS-catalyst (0.1 eq.) and BH_3_·Me_2_S (1 eq.) provided the diastereoisomeric mixture **5**/**6** (50:50), determined by ^1^H NMR. BH_3_·Me_2_S reduction of amino-β-lactam ketone **4** (1:1 eq. ratio) had no effect on the stereoselectivity, providing mixture **5**/**6** (50:50). The absence of stereoselectivity in the reduction of amino-β-lactam ketone **4** was probably due to nitrogen proximity to the keto group and the ability of borane to form a complex with amine, which allowed a direct hydrogen delivery to the keto group without participation of a chiral catalyst [22], [23]. Addition of BH_3_·Me_2_S (2 eq.) to CBS-catalyst (0.1 eq.) did not result in improvement of stereoselectivity in keto group reduction of **4** either. Improvement of stereoselectivity was accomplished with the complex formation between CBS-catalyst and BH_3_·Me_2_S, (1:1 eq. ratio), followed by dropwise addition of **4**. Reaction with (*R*)-CBS-catalyst provided a diastereoisomeric mixture of amino alcohols **5**/**6** (70:30) at −20 °C, whereas (*S*)-CBS-catalyst afforded **6**/**5** (85:15) (Fig. 4). Lowering the temperature to −80 °C did not improve the stereoselectivity of the reduction. Recrystallization of **6**/**5** (85:15) provided pure amino alcohol **6**.

**Scheme 2:**
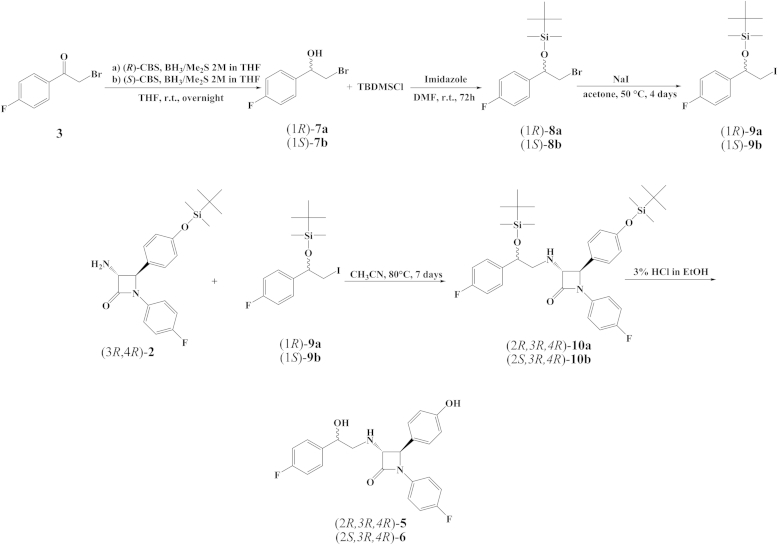
*N*-alkylation of *trans*-amino-β-lactam (3*R*,4*R*)-**2** with (1*R*)-**9a** and (1*S*)-**9b** for the preparation of amino alcohols (2*R*,3*R*,4*R*)-**5** and (2*S*,3*R*,4*R*)-**6**.

Commercially available ketone **3** was reduced following the original protocol for CBS-reduction developed by Corey et al. and yielded alcohols **7a** and **7b** in >99% ee (Scheme 2) [19]. Protection of the OH group in **7a**,**b** with TBDMSCl was carried out in DMF in the presence of imidazole [24] to afford OTBDMS bromo derivatives **8a** and **8b**. Exchange of bromine in **8a**,**b** with iodine (in the presence of NaI in acetone at 55 °C) yielded OTBDMS iodo derivatives **9a** and **9b**. Iodo-bromo exchange proceeded very slowly, providing the mixtures of **9a** or **9b** and unreacted **8a** or **8b**, respectively, in ratio 93:7 and 96% yield after 4 days. This mixture was used in the *N*-alkylation reaction of amino-β-lactam **2** without further purification. The reaction proceeded for 7 days in CH_3_CN to afford silyl intermediates **10a** and **10b** with a moderate yield (20%). Silyl intermediates **10a**,**b** were further deprotected with 3% HCl in ethanol to produce ezetimibe bioisosteres **5** and **6** (Scheme 2).

#### 2.1.2 Molecular and crystal structure

The crystal structure of (3*R*,4*R*)-3-[(2*S*)-2-(4-fluorophenyl)-2-hydroxyethylamino]-1-(4-fluorophenyl)-4-(4-hydroxyphenyl)azetidin-2-one (2*S*,3*R*,4*R*)-**6** was determined to establish unambiguously both absolute and relative configurations at the stereogenic centres C17 [25] and N2.

**Fig. 5:**
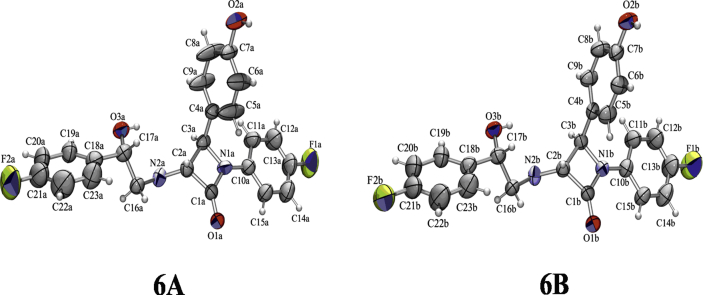
ORTEP-3 [26] drawing of **6A** and **6B** symmetry-indepentent molecules of (2*S*,3*R*,4*R*)-**6**. Displacement ellipsoids are drawn for the probability of 50% and hydrogen atoms are shown as spheres of arbitrary radii.

**Fig. 6:**
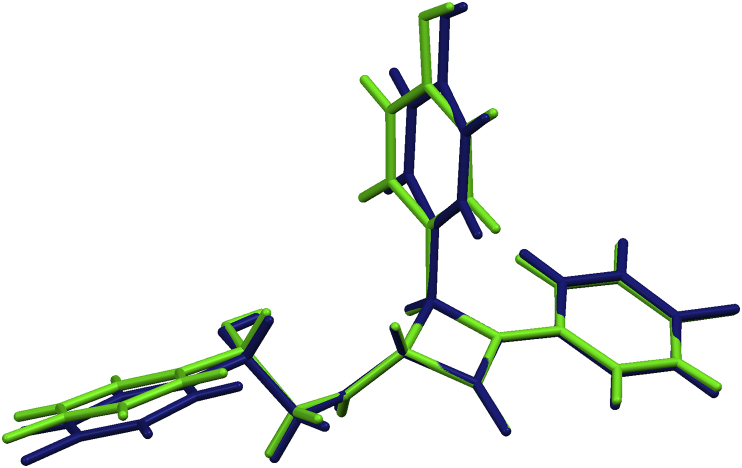
Overlay of molecules **6A** (black) and **6B** (grey).

Two symmetry independent molecules of **6**, related by a pseudo-twofold rotation axis, are present in the crystal structure designated as **6A** and **6B** (Fig. 5); they are homochiral and of similar conformations (Fig. 6). Configuration at the stereogenic centre C17 was assigned to be *S* in both **6A** and **6B**, according to the known *R*-configurations at the stereogenic centres C2 and C3.

**Fig. 7:**
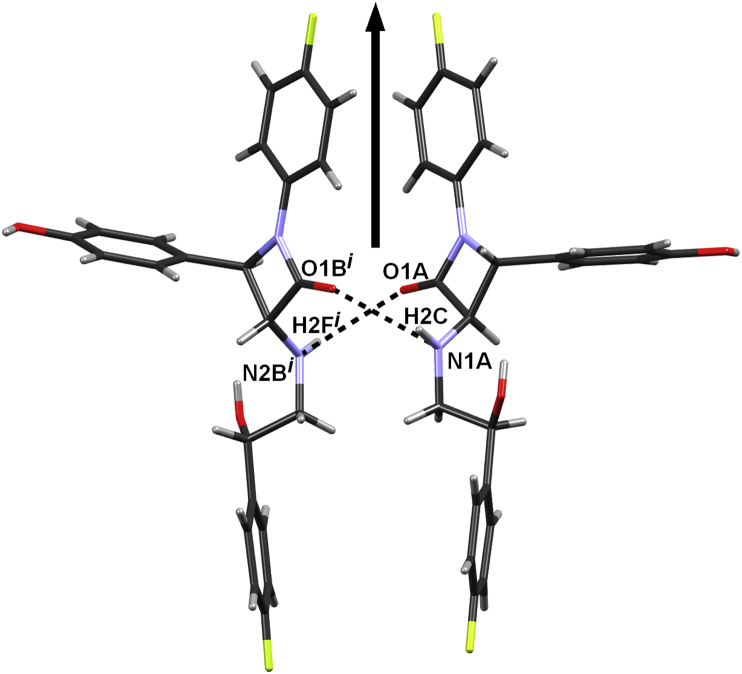
The two independent molecules **6A** and **6B** are connected by hydrogen bonds into a dimer of pseudo-*C*_2_ symmetry. The pseudo-*C*_2_ axis is approximately parallel to the crystallographic axis *a*, marked by an arrow (↑). Symmetry operator *i*) 1 + *x*, *y*, *z*.

**Fig. 8:**
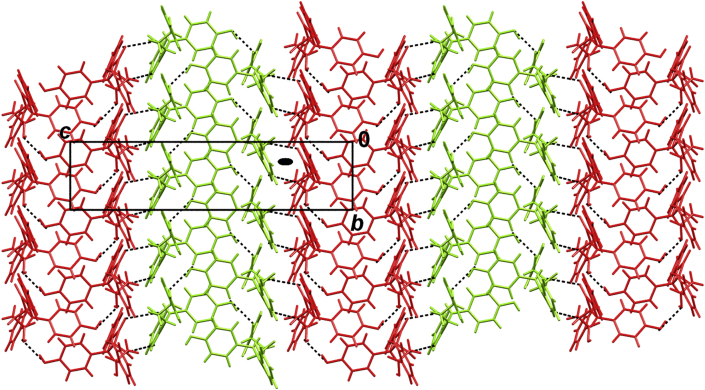
Hydrogen-bonded layer in (2*S*,3*R*,4*R*)-**6**. Molecules **6A** (red) and **6B** (green). Position of the pseudo-*C*_2_ axis has been indicated by the black oval. Approximate position of the pseudo axis is *y* ≈ 0.20; *z* ≈ 0.26.

Molecules **6A** and **6B** are linked by a pair of N–H⋯O=C hydrogen bonds into *C*_2_-symmetric dimers (Fig. 7, Table 1). Each molecule has two hydroxy groups, which act as proton donors toward symmetry-equivalent molecules; one toward a carbonyl and one toward another hydroxy group, giving a total of four O–H···O hydrogen bonds (Table 1) that link the dimers into layers parallel to (011). There are only dispersion interactions between the layers (Fig. 8).

**Table 1:** Geometric parameters of the hydrogen bonds in (2*S*,3*R*,4*R*)-**6**.

	*D*–H/Å	H⋯*A*/Å	*D*⋯*A*/Å	*D*–H⋯*A*/°	Symm. op. on *A*
N2A–H2C⋯O1B	0.86(3)	2.11(3)	2.962(7)	172(4)	1+*x*, *y*, *z*
N2B–H2F⋯O1A	0.90	2.16	3.019(6)	159	−1+*x*, *y*, *z*
O2A–H2B⋯O3A	0.82	1.91	2.692(7)	159	2–*x*, 1/2 + *y*, 1–*z*
O2B–H2E⋯O3B	0.82	1.91	2.702(6)	162	–*x*, 1/2 + *y*, –*z*
O3A–H3B⋯O1A	0.82	2.75	3.334(6)	129	*x*, −1+*y*, *z*
O3B–H3D⋯O1B	0.82	2.57	3.185(6)	133	*x*, 1 + *y*, *z*
C2A–H2A⋯O2A	0.98	2.42	3.365(8)	161	2–*x*, 1/2 + *y*, 1–*z*
C2B–H2B⋯O2B	0.98	2.48	3.388(7)	155	–*x*, 1/2 + *y*, –*z*
C12A–H12A⋯F2A	0.93	2.35	3.280(13)	173	−1+*x*, *y*, *z*

#### 2.1.3 pK_a_ values

**Fig. 9:**
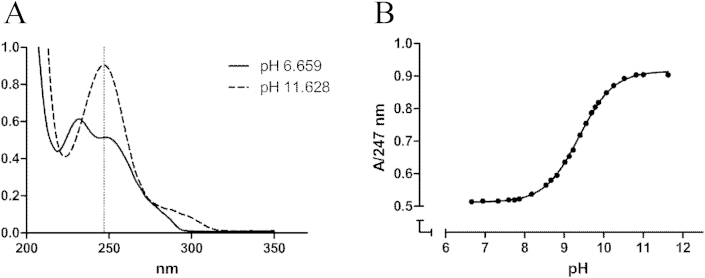
(**A**) UV spectra of **5** at pH 6.6 and pH 11 and (**B**) absorbance of **5** at *λ* 247 nm and pH 6–12.

We determined p*K*_a_ values of the amino alcohols **5** (Fig. 9) and **6** (data not shown) using spectrophotometric titration. We measured an increase of the absorbance intensity at *λ* 247 nm by the addition of NaOH in the pH range 6–12 (Fig. 9A). p*K*_a_ values calculated from the obtained sigmoidal curves for amino alcohol **5** (Fig. 9B) revealed that the p*K*_a_ value was 9.35. We got the same p*K*_a_ value for amino alcohol 6 (data not shown), indicating that both compounds are present in the form of NH_4_^+^ in the blood and small intestine.

### 2.2 Biochemical section

#### 2.2.1 Cytotoxicity measurement

Cytotoxicity of amino alcohols **5**, **6** and their diastereoisomeric mixture **5**/**6** was analyzed using MTT cell proliferation assay and the LC_50_ values were determined in MDCKII wild type, hNPC1L1/MDCKII, and HepG2 cells (Table 2). MDCKII cells stably expressing human Niemann-Pick C1-like protein 1 (hNPC1L1/MDCKII) (Figure S1) are a pharmacologically validated system for investigating NPC1L1-mediated cholesterol uptake [27]. The LC_50_ values were higher than 100 μM and considered nontoxic in all three cell lines. LC_50_ values for ezetimibe **1** were 62.29 μM in hNPC1L1/MDCKII and 69.74 μM in HepG2 cells. In MDCKII wild type cells, ezetimibe **1** showed no toxicity (Table 2). In addition, we tested the *in vitro* cytotoxicity of ezetimibe **1** and compounds **5**, **6**, and **5**/**6** (70:30) in combination with micelles and found all LC_50_ values to be above 100 μM (Figure S2).

**Table 2:** *In vitro* cytotoxicity assay expressing LC_50_ (μM).

Compound	MDCKII	hNPC1L1/MDCKII	HepG2
**1**	>100	62,29	69,74
**5**	>100	>100	>100
**6**	>100	>100	>100
**5**/**6** (70:30)	>100	>100	>100

#### 2.2.2 *In vitro* activity

**Fig. 10:**
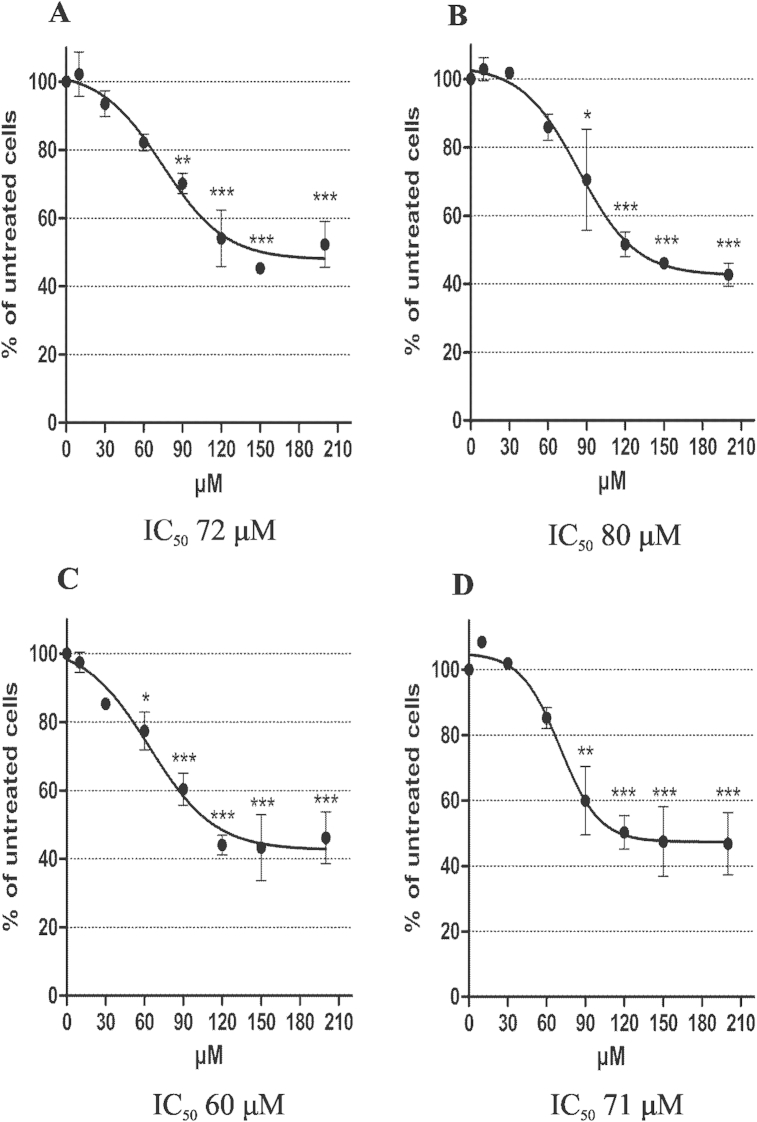
Cholesterol uptake inhibition of novel β-lactam amino alcohols **5** and **6** and their diastereoisomeric mixtures **5**/**6** (70:30) and **6**/**5** (85/15). Cholesterol uptake was determined in hNPC1L1/MDCKII cells. Cells were treated with indicated concentrations of (**A**) **5**, (**B**) **6**, and their diastereoismeric mixtures (**C**) **5**/**6** (70:30) and (**D**) **6**/**5** (85:15) for 1 h. The results are expressed as percentage of inhibition compared to untreated cells and represent mean ± S.E.M. of three independent experiments. IC_50_ values were determined for each compound or mixture. *p < 0.05, **p < 0.01, ***p < 0.001 determined by one-way ANOVA followed by Dunnett's test.

First we tested ezetimibe analogs **5** and **6** and their diastereoisomeric mixtures **5**/**6** (70:30) and **6**/**5** (85:15) for their ability to inhibit cholesterol uptake. In MDCKII wildtype cells, ezetimibe **1** had no effect, but inhibited cholesterol uptake in hNPC1L1/MDCKII cells (Figure S3). When hNPC1L1/MDCKII were treated with **5** (Fig. 10A), **6** (Fig. 10B), and their diastereoisomeric mixtures **5**/**6** (Fig. 10C) and **6**/**5** (Fig. 10D), we observed 50–55% inhibition of cholesterol uptake, reaching its maximum at 120 μM concentration without a significant difference between the compounds. IC_50_ values were in the range of 60–80 μM (Fig. 10). These results show that the novel ezetimibe bioisosteres **5**, **6** and their diastereoisomeric mixtures **5**/**6** and **6**/**5** are potent inhibitors of cholesterol uptake *in vitro*.

#### 2.2.3 *In vivo* activity

*In vivo*, we first determined the consequences of ezetimibe **1** and amino alcohols **5** and **6** on cholesterol absorption. We therefore gavaged mice with 2 μCi [^3^H]cholesterol and 10 mg/kg/day of each compound or vehicle, and radioactivity was measured in plasma, liver, and three equal parts of the intestine (duodenum, jejunum, ileum). Percentage of [^3^H]cholesterol reduction is presented relative to vehicle, respectively. Both **5** and **6** showed significant inhibition of cholesterol absorption (Table 3). Treatment of mice with either **5** and **6** resulted in reductions of [^3^H]cholesterol in plasma by 50% and 32%, respectively. Radioactivities in the liver were decreased by 44% and 47%, respectively, and were comparable to ezetimibe **1**. Amino alcohol **6** markedly lowered [^3^H]cholesterol in the small intestine with highest inhibition in the ileum by 58–60%, whereas amino alcohol **5** reduced radioactivity in the small intestine by ∼22% without reaching statistical significance in either part of the intestine. We therefore increased the dose to 20 mg/kg/day and determined inhibition of cholesterol absorption. The inhibition in duodenum and jejunum was similar to the lower dose, whereas in ileum it raised from 21% to 41% (Table 3). Decreased cholesterol absorption of **5** in the small intestine compared to ezetimibe **1** might be explained by the fact that 2-azetidinones are moderate acyl-coenzyme A:cholesterol acyltransferase inhibitors and their level of activity is highly structure-dependent [28]. It might therefore be speculated that acyl-coenzyme A:cholesterol acyltransferase activity of amino alcohols **5** and **6** and their diastereoisomeric mixtures is altered compared to ezetimibe **1**. The effect of the diastereoisomeric mixture **5**/**6** (70:30) on cholesterol absorption was comparable to ezetimibe **1** in plasma and ileum, lower in duodenum and jejunum, and higher in the liver (Table 3).

**Table 3:** *In vivo* inhibition (%) of cholesterol absorption by compounds **1**, **5**, **6**, and the diastereoisomeric mixture **5**/**6** (**70**:**30**).

Compound	Plasma	Duodenum	Jejunum	Ileum	Liver
**1**^a^	60 ± 6***	65 ± 1***	61 ± 2***	47 ± 3*	52 ± 3***
**5**^a^	50 ± 8***	23 ± 18	23 ± 13	21 ± 20	44 ± 6***
**5**^b^	52 ± 7***	18 ± 14	19 ± 16	41 ± 12*	33 ± 6***
**6**^a^	32 ± 5***	43 ± 11**	37 ± 11**	58 ± 8**	47 ± 0***
**6**^b^	38 ± 11***	35 ± 9*	26 ± 8	60 ± 9**	49 ± 10***
**5**/**6**^c^ (70:30)	64 ± 7***	49 ± 7**	48 ± 9***	41 ± 21	70 ± 5***

^a,b^5 mice or ^c^4 mice per group were gavaged with compounds ^a^10 mg/kg/day or ^b,c^20 mg/kg/day for 2 days.

*p < 0.05, **p < 0.01, ***p < 0.001 determined by one-way ANOVA followed by Dunnett's test.

## 3 Conclusion

This report demonstrates that we have successfully synthesized two novel ezetimibe bioisosteres **5**, **6** and their diastereoisomeric mixtures **5**/**6** (70:30) and **6**/**5** (85:15) from enantiomerically pure *trans*-(3*R*,4*R*)-amino-β-lactam **2**. Crystal structure of **6** was determined to establish unambiguously both absolute and relative configurations at the new stereogenic centre C17 and were assigned to be *S*. Both diastereoisomeres **5** and **6** as well as their diastereoisomeric mixture **5**/**6** showed significant cholesterol absorption inhibitory activity both *in vitro* and *in vivo*. Based on our data and the p*K*_a_ value for **5** and **6** being 9.35, indicating that both compounds are present in the form of NH_4_^+^ in the blood and small intestine, other diastereoisomeric mixtures (e.g. **6**/**5** (85:15)) may exhibit similar *in vivo* results as **5**/**6** (70:30). Results from this study implicate a therapeutic potential of these novel compounds to reduce cholesterol plasma concentrations and improve CHD.

## 4 Experimental protocols

### 4.1 Materials and methods

All commercial reagent grade chemicals and solvents were used without further treatment. Melting points were determined on a Reichert Thermovar 7905 apparatus and are not corrected. The IR spectra were recorded on a PerkinElmer Spectrum RX I FT-IR System spectrometer (KBr pellets technique) (PerkinElmer Instruments, Norwalk, CT, USA). The ^1^H and ^13^C NMR spectra (in CDCl_3_ and DMSO-*d*_*6*_ at RT) were measured on a Bruker AV 300 and/or AV 600 spectrometer (Bruker BioSpin GmbH., Rheinstetten, Germany), *δ* is given in ppm relative to tetramethylsilane as an internal reference. Microanalysis was performed on a PE 2400 Series II CHNS/O Analyzer (PerkinElmer Instruments, Shelton, CT, USA). Optical rotations: Optical Activity Automatic Polarimeter AA-10 in 1 dm cell; *c* in g/100 mL (Optical Activity Ltd., Ramsey, England). Column chromatography on silica gel, 70–230 mesh, 60 Å (Sigma–Aldrich, St. Louis, MO, USA or Acros-Organics, New Jersey, USA) was performed at RT. Thin layer chromatography was carried out on TLC aluminium sheets, 20 × 20 cm, silica gel 60 F_254_ (Merck, Darmstadt, Germany). RP-HPLC analysis was performed on HPLC system (Knauer GmbH., Berlin, Germany) supplied with UV/VIS WellChrom Diode Array Detector K-2800. UV/VIS measurements were performed on a T80+ spectrophotometer (PG Instruments Limited, England). Samples for HR-MS analysis were resuspended in 5 μl of THAP/DAC matrix and 1 μl was spotted onto a MALDI plate. Mass spectra were obtained on a matrix-assisted laser desorption/ionization-time-of-flight MALDI-TOF/TOF mass spectrometer (4800 Plus MALDI-TOF/TOF Analyzer, Applied Biosystems, Foster City, CA, USA) equipped with Nd:YAG laser operating at 355 nm with firing rate 200 Hz in the positive ion reflector mode. 1600 shots per spectrum were taken covering mass range 100–1000 Da, focus mass 500 Da and delay time 100 ns.

### 4.2 Crystal structure determination

Crystals of amino alcohol (2*S*,3*R*,4*R*)-**6** suitable for data collection were grown from ethyl acetate and hexane by vapour diffusion. The selected crystal was a needle with dimensions 0.10 × 0.03 × 0.02 mm^3^.

Single crystal measurement was performed on an Oxford Diffraction Xcalibur Nova R (microfocus Cu tube, Oxford Diffraction, U.K.) at room temperature [293(2) K]. Program package CrysAlis PRO [29] was used for data reduction. The structure was solved refined with SHELXS97 [30]. The model was refined using the full-matrix least squares refinement; all non-hydrogen atoms were refined anisotropically. Hydrogen atoms bound to C and O were modelled as riding entities using the AFIX command. Hydrogen atoms bound to N2A and N2B could not be located from the electron density map due to the data poor quality. However, it can be assumed that they are directed towards the nearest proton acceptor: the carbonyl oxygen of the neighbouring molecule. Therefore, they were generated at the appropriate positions and refined using appropriate restraints (Table 1).

Configuration of novel stereogenic centre C17 was established relative to the configurations of the known stereogenic centres C2 and C3. Friedel pairs were therefore not measured.

The structure comprises relatively large cavities filled by disordered water molecules. Due to a very low electron density and low data quality (due to small size of the crystal) the disordered water was tentatively modelled as four isotropic oxygen atoms (hydrogens could not have been located) with occupancy of 0.5.

Molecular geometry calculations were performed by PLATON [31], and molecular graphics were prepared using ORTEP-3 [26], and CCDC-Mercury [32]. Crystallographic and refinement data for amino alcohol (2*S*,3*R*,4*R*)-**6** reported in this paper are shown in Table 4.

**Table 4:** Crystallographic data collection and structure refinement details for amino alcohol (2*S*,3*R*,4*R*)-**6**.

Compound	**6**
Empirical formula	C_23_H_23_F_2_N_2_O_4_
Formula wt./g mol^−1^	426.41
Crystal dimensions/mm	0.10 × 0.03 × 0.02
Space group	*P* 2_1_
*a*/Å	16.3185(9)
*b*/Å	6.0148(3)
*c*/Å	25.0538(14)
*α*/°	90
*β*/°	95.139(5)
*γ*/°	90
Z	4
*V*/Å^3^	2449.2(2)
*D*_calc_/g cm^−3^	1.156
*μ*/mm^−1^	0.758
Θ range/°	3.11–76.27
*T*/K	293(2)
Radiation wavelength	1.54179 (Cu*K*α)
Diffractometer type	Xcalibur Nova
Range of *h*, *k*, *l*	−20 < *h* < 18; −7 < *k* < 6; −30 < *l* < 31
Reflections collected	12653
Independent reflections	6976
Observed reflections (*I* ≥ 2*σ*)	5385
Absorption correction	Multi-scan
*R*_int_	0.0429
*R* (*F*)	0.1013
*R*_*w*_ (*F*^2^)	0.3305
Goodness of fit	1.326
H atom treatment	Mixed
No. of parameters	558
No. of restraints	39
Δ*ρ*_max_, Δ*ρ*_min_ (eÅ^−3^)	0.646; −0.388

Supplementary crystallographic data for this paper can be obtained free of charge *via* www.ccdc.cam.ac.uk/conts/retrieving.html (or from the Cambridge Crystallographic Data Centre, 12, Union Road, Cambridge CB2 1EZ, UK; fax: (+)44 1223 336033; or deposit@ccdc.cam.ac.uk). CCDC-979536 contains the supplementary crystallographic data for this paper.

### 4.3 Synthesis of amino-β-lactam derivatives

#### 4.3.1 (3*R*,4*R*)-3-amino-1-(4-fluorophenyl)-4-(4-(*t*-butyldimethylsilyloxy)phenyl)azetidin-2-one (**2**)

Amino-β-lactam **2** was synthesized following the procedure described by Ojima et al. [16] and Habuš et al. [17] and purified by a silica gel column chromatography (hexane/ethyl acetate 9:1, gradually increasing the content of ethyl acetate in eluent to pure ethyl acetate) and obtained as yellow oil (1.3 g, 43%). [α]_D_^20^ = −26 (*c* = 1, EtOAc). FT-IR (KBr) cm^−1^: 2931, 2858, 1739, 1609, 1510, 1390, 1266, 913, 834, 513. ^1^H NMR (300 MHz, CDCl_3_): 0.19 (s, 6H, Si-(C*H*_3_)_2_), 0.97 (s, 9H, C-(C*H*_3_)_3_), 1.81 (bs, 2H, N*H*_2_), 4.05 (d, 1H, *J* = 2.2 Hz, C4 β-lactam), 4.58 (d, 1H, *J* = 2.1 Hz, C3 β-lactam), 6.83 (d, 2H, *J* = 8.5 Hz, Ar–*H*), 6.92 (t, 2H, *J*_1,2_ = 8.7 Hz, Ar–*H*), 7.18 (d, 2H, *J* = 8.5 Hz, Ar–*H*), 7.23–7.26 (m, 2H, Ar–*H*); ^13^C NMR (150 MHz, CDCl_3_): −4.27 (Si-(*C*H_3_)_2_), 18.32 (*C*-(CH_3_)_3_), 25.75 (C-(*C*H_3_)_3_), 66.69 (C4 β-lactam), 70.17 (C3 β-lactam), 115.95 (d, *J* = 22.8 Hz, 4-F-*C*_6_H_4_), 118.98 (d, *J* = 7.8 Hz, 4-F-*C*_6_H_4_), 120.86 (4-OTBDMS-*C*_6_H_4_), 127.31 (4-OTBDMS-*C*_6_H_4_), 129.18 (4-OTBDMS-*C*_6_H_4_), 133.83 (d, *J* = 2.2 Hz, 4-F-*C*_6_H_4_), 156.25 (4-OTBDMS-*C*_6_H_4_), 159.26 (d, *J* = 243.9 Hz, 4-F-*C*_6_H_4_), 168.02 (C=O, β-lactam). HRMS for C_21_H_27_FN_2_O_2_Si (*M*_r_ = 386.53518): calcd. m/z [M+H^+^] 387.1898, found 387.1901.

#### 4.3.2 2-[(3*R*,4*R*)-1-(4-fluorophenyl)-4-(4-(*t*-butyldimethylsilyloxy)phenyl)-2-oxo-3-azetidinylamino]-1-(4-fluorophenyl)ethan-1-one (**4**)

To a solution of **2** (159 mg, 0.41 mmol) in a mixture of anhydrous THF and DMF (9:1, 7.8 mL), 2-bromo-1-(4-fluorophenyl)ethan-1-one **3** (98 mg, 0.45 mmol), sodium iodide (68 mg, 0.45 mmol) and Et_3_N (114 μL, 0.82 mmol) were added. The reaction mixture was stirred at room temperature for 3 h after which solvent was evaporated to dryness. Distilled water (10 mL) was added and the product extracted with ethyl acetate (3 × 20 mL). Collected organic layers were dried over Na_2_SO_4_ and solvent evaporated to dryness. **4** was purified by a silica gel column chromatography (hexane/ethyl acetate 2:1) and obtained as light brown oil (99 mg, 46%). [α]_D_^20^ = +20 (*c* = 1, EtOAc). FT-IR (KBr) cm^−1^: 3332, 2931, 1748, 1693, 1600, 1506, 1385, 1260, 1156, 913, 834, 515. ^1^H NMR (600 MHz, CDCl_3_): 0.18 (s, 6H, Si-(C*H*_3_)_2_), 0.96 (s, 9H, C-(C*H*_3_)_3_), 1.60 (bs, 1H, N*H*), 4.09 (d, 1H, *J* = 2.1 Hz, C4 β-lactam), 4.26 and 4.34 (ABq, 2H, *J*_AB_ = 18.5 Hz, C(=O)–C*H*_2_–NH), 4.80 (d, 1H, *J* = 1.9 Hz, C3 β-lactam), 6.80 (d, 2H, *J* = 8.5 Hz, Ar–*H*), 6.93 (t, 2H, *J*_1,2_ = 8.6 Hz, Ar–*H*), 7.12–7.16 (m, 4H, Ar–*H*), 7.25–7.27 (m, 2H, Ar–*H*), 7.94–7.97 (m, 2H, Ar–*H*); ^13^C NMR (75 MHz, CDCl_3_): −4.28 (Si-(*C*H_3_)_2_), 18.30 (*C*-(CH_3_)_3_), 25.74 (C-(*C*H_3_)_3_), 53.00 (C(=O)–*C*H_2_–NH), 63.79 (C4 β-lactam), 75.44 (C3 β-lactam), 115.98 (d, *J* = 22.6 Hz, 4-F-*C*_6_H_4_), 116.16 (d, *J* = 21.7 Hz, 4-F-*C*_6_H_4_), 119.06 (d, *J* = 7.9 Hz, 4-F-*C*_6_H_4_), 120.87 (4-OTBDMS-*C*_6_H_4_), 127.30 (4-OTBDMS-*C*_6_H_4_), 129.12 (4-OTBDMS-*C*_6_H_4_), 130.62 (d, *J* = 9.4 Hz, 4-F-*C*_6_H_4_), 131.57 (d, *J* = 3.3 Hz, 4-F-*C*_6_H_4_), 133.68 (d, *J* = 2.8 Hz, 4-F-*C*_6_H_4_), 156.19 (4-OTBDMS-*C*_6_H_4_), 159.31 (d, *J* = 243.9 Hz, 4-F-*C*_6_H_4_), 166.17 (C=O, β-lactam), 166.23 (d, *J* = 254.8 Hz, 4-F-*C*_6_H_4_), 195.58 (C=O). HRMS for C_29_H_32_F_2_N_2_O_3_Si (*M*_r_ = 522.65829): calcd. *m/z* [M+K^+^] 561.1782, found 561.1788.

#### 4.3.3 (3*R*,4*R*)-3-[(2*R*/*S*)-2-(4-fluorophenyl)-2-hydroxyethylamino]-1-(4-fluorophenyl)-4-(4-hydroxyphenyl)azetidin-2-one (**5**/**6**, 70:30)

The reaction was carried out in dry conditions under argon atmosphere. To a solution of (*R*)-(+)-2-methyl-CBS-oxazaborolidine catalyst (251 mg, 0.91 mmol) in anhydrous THF (2 mL), 2M THF solution of BH_3_·Me_2_S (454 μL, 0.91 mmol) was added and the mixture stirred at room temperature for 10 min. The temperature was then lowered to −20 °C and a solution of **4** (474 mg, 0.91 mmol) in anhydrous THF (5 mL) added dropwise. After 24 h the reaction mixture was warmed to room temperature and quenched with methanol. The solution was concentrated *in vacuo*, distilled water (15 mL) added and the product extracted with ethyl acetate (3 × 40 mL). Collected organic layers were dried over Na_2_SO_4_ and solvent evaporated to dryness. Crude product was dissolved in ethanol (22 mL) and 1 M HCl (5 mL) added. Reaction mixture was stirred at room temperature overnight, solvent evaporated to dryness and crude product purified by a silica gel column chromatography (hexane/ethyl acetate 1:3) to afford diastereoisomeric mixture **5**/**6** (70:30) (283 mg, 76%). mp 125–127 °C. FT-IR (KBr) cm^−1^: 3341, 1736, 1615, 1601, 1509, 1390, 1223, 1154, 1101, 832. ^1^H NMR (600 MHz, DMSO-*d*_*6*_): 2.72–2.83 (m, 2H, CH(OH)–C*H*_2_–NH, **5**/**6**), 2.93 (bs, 1H, CH(OH)–CH_2_–N*H*, **5**/**6**), 3.97 (s, 1H, C4 β-lactam, **5**/**6**), 4.55–4.57 (m, 1H, C*H*(OH)–CH_2_–NH, **6**), 4.59–4.62 (m, 1H, C*H*(OH)–CH_2_–NH, **5**), 4.83 (d, 1H, *J* = 2.0 Hz, C3 β-lactam, **5**/**6**), 5.30 (d, 1H, *J* = 4.3 Hz, CH(O*H*)–CH_2_–NH, **5**), 5.33 (d, 1H, *J* = 4.3 Hz, CH(O*H*)–CH_2_–NH, **6**), 6.73–6.75 (m, 2H, Ar–*H*, **5**/**6**), 7.09–7.23 (m, 8H, Ar–*H*, **5**/**6**), 7.30–7.36 (m, 2H, Ar–*H*, **5**/**6**), 9.43 (s, 1H, Ar–O*H*, **5**/**6**); ^13^C NMR (150 MHz, DMSO-*d*_*6*_): 54.53 (CH(OH)–*C*H_2_–NH, **5**), 54.78 (CH(OH)–*C*H_2_–NH, **6**), 62.36 (C4 β-lactam, **5**), 62.58 (C4 β-lactam, **6**), 71.43 (*C*H(OH)–CH_2_–NH, **5**), 71.61 (*C*H(OH)–CH_2_–NH, **6**), 75.43 (C3 β-lactam, **5**), 75.63 (C3 β-lactam, **6**), 114.60 (d, *J* = 20.9 Hz, 4-F-*C*_6_H_4_, **5**), 114.62 (d, *J* = 21.2 Hz, 4-F-*C*_6_H_4_, **6**), 115.68 (4-OH-*C*_6_H_4_, **5**/**6**), 115.79 (d, *J* = 22.7 Hz, 4-F-*C*_6_H_4_, **5**/**6**), 118.68 (d, *J* = 7.7 Hz, 4-F-*C*_6_H_4_, **5**/**6**), 127.40 (4-OH-*C*_6_H_4_, **6**), 127.48 (4-OH-*C*_6_H_4_, **5**), 127.52 (4-OH-*C*_6_H_4_, **5**), 127.58 (4-OH-*C*_6_H_4_, **6**), 127.85 (d, *J* = 7.8 Hz, 4-F-*C*_6_H_4_, **5**/**6**), 133.86 (d, *J* = 1.9 Hz, 4-F-*C*_6_H_4_, **5**/**6**), 140.33 (d, *J* = 2.7 Hz, 4-F-*C*_6_H_4_, **6**), 140.36 (d, *J* = 2.8 Hz, 4-F-*C*_6_H_4_, **5**), 157.34 (4-OH-*C*_6_H_4_, **5**/**6**), 158.12 (d, *J* = 240.3 Hz, 4-F-*C*_6_H_4_, **5**/**6**), 161.19 (d, *J* = 242.4 Hz, 4-F-*C*_6_H_4_, **5**/**6**), 167.06 (C=O, β-lactam, **5**), 167.08 (C=O, β-lactam, **6**). HRMS for C_23_H_20_F_2_N_2_O_3_ (*M*_r_ = 410.41331): calcd. *m/z* [M+H^+^] 411.1515, found 411.1513.

#### 4.3.4 (3*R*,4*R*)-3-[(2*S*/*R*)-2-(4-fluorophenyl)-2-hydroxyethylamino]-1-(4-fluorophenyl)-4-(4-hydroxyphenyl)azetidin-2-one (**6**/**5**, 85:15)

Reaction was carried out following the procedure for **5**/**6**. To a solution of (*S*)-(−)-2-methyl-CBS-oxazaborolidine catalyst (275 mg, 0.99 mmol) in anhydrous THF (2 mL), 2 M THF solution of BH_3_·Me_2_S (496 μL, 0.99 mmol) and **4** (519 mg, 0.99 mmol) in anhydrous THF (5 mL) were added. Diastereoisomeric mixture **6**/**5** (85:15) (278 mg, 68%) was obtained. mp 118–120 °C.

#### 4.3.5 (3*R*,4*R*)-3-[(2*S*)-2-(4-fluorophenyl)-2-hydroxyethylamino]-1-(4-fluorophenyl)-4-(4-hydroxyphenyl)azetidin-2-one (**6**)

Amino alcohol **6** was obtained (111 mg, 25%) by recrystallization of diastereoisomeric mixture **6**/**5** (85:15) in CH_2_Cl_2_. mp 118–120 °C. [α]_D_^20^ = +9 (*c* = 1, EtOAc). FT-IR (KBr) cm^−1^: 3344, 1723, 1615, 1510, 1394, 1233, 1155, 829. ^1^H NMR (300 MHz, DMSO-*d*_*6*_): 2.70–2.86 (m, 2H, CH(OH)–C*H*_2_–NH), 3.08 (bs, 1H, CH(OH)–CH_2_–N*H*), 3.97 (d, 1H, *J* = 1.9 Hz, C4 β-lactam), 4.54–4.59 (m, 1H, C*H*(OH)–CH_2_–NH), 4.84 (d, 1H, *J* = 1.9 Hz, C3 β-lactam), 5.38 (d, 1H, *J* = 4.3 Hz, CH(O*H*)–CH_2_–NH), 6.75 (d, 2H, *J* = 8.5 Hz, Ar–*H*), 7.07–7.25 (m, 8H, Ar–*H*), 7.30–7.34 (m, 2H, Ar–*H*), 9.48 (s, 1H, Ar–O*H*); ^13^C NMR (75 MHz, DMSO-*d*_*6*_): 54.76 (CH(OH)–*C*H_2_–NH), 62.57 (C4 β-lactam), 71.56 (*C*H(OH)–CH_2_–NH), 75.57 (C3 β-lactam), 114.58 (d, *J* = 21.1 Hz, 4-F-*C*_6_H_4_), 115.65 (4-OH-*C*_6_H_4_), 115.74 (d, *J* = 22.6 Hz, 4-F-*C*_6_H_4_), 118.63 (d, *J* = 8.0 Hz, 4-F-*C*_6_H_4_), 127.34 (4-OH-*C*_6_H_4_), 127.52 (4-OH-*C*_6_H_4_), 127.78 (d, *J* = 8.1 Hz, 4-F-*C*_6_H_4_), 133.85 (d, *J* = 2.5 Hz, 4-F-*C*_6_H_4_), 140.25 (d, *J* = 2.8 Hz, 4-F-*C*_6_H_4_), 157.33 (4-OH-*C*_6_H_4_), 158.08 (d, *J* = 240.7 Hz, 4-F-*C*_6_H_4_), 161.17 (d, *J* = 242.2 Hz, 4-F-*C*_6_H_4_), 167.02 (C=O, β-lactam). HRMS for C_23_H_20_F_2_N_2_O_3_ (*M*_r_ = 410.41331): calcd. m/z [M+H^+^] 411.1515, found 411.1522.

#### 4.3.6 (1*R*)-2-bromo-1-(4-fluorophenyl)ethan-1-ol (7a) and (1*S*)-2-bromo-1-(4-fluorophenyl)ethan-1-ol (**7b**)

Reaction was carried out in dry conditions under argon atmosphere. For the synthesis of **7a**, 2 M THF solution of BH_3_·Me_2_S (1.15 mL, 2.30 mmol) was added to a solution of (*R*)-(+)-2-methyl-CBS-oxazaborolidine catalyst (64 mg, 0.23 mmol) in anhydrous THF (2 mL). The mixture was stirred for 10 min and a solution of 2-bromo-1-(4-fluorophenyl)ethan-1-one **3** (500 mg, 2.30 mmol) in anhydrous THF (4 mL) was added dropwise. Reaction proceeded at room temperature overnight. Reaction mixture was quenched with methanol, solvent evaporated to dryness and distilled water (15 mL) added. The product was extracted with CH_2_Cl_2_ (3 × 30 mL), collected organic layers dried over Na_2_SO_4_ and solvent evaporated to dryness. **7a** was purified by a silica gel column chromatography (hexane/ethyl acetate 6:1) and obtained as colourless oil (503 mg, 100%). The ee>99% of **7a** was determined by chiral HPLC (200/4 Nucleodex beta-PM Column, Macherey–Nagel, Germany, Method: 0.1% TEAA in H_2_O: methanol 55: 45, 30 min, flow 0.7 mL/min, *λ* = 254 nm, retention time 19.5 min). [α]_D_^20^ = −28 (*c* = 1, EtOAc). **7b** was synthesized following the same procedure using (*S*)-(−)-2-methyl-CBS-oxazaborolidine catalyst. The ee>99% was determined by chiral HPLC (retention time 18.0 min). [α]_D_^20^ = +28 (*c* = 1, EtOAc). FT-IR (KBr) cm^−1^: 3401, 2963, 2897, 1896, 1605, 1513, 1306, 1224, 1158, 1067, 992, 838, 779, 645, 556, 523. ^1^H NMR (300 MHz, CDCl_3_): 2.75 (bs, 1H, CH(O*H*)–CH_2_), 3.46–3.61 (m, 2H, CH(OH)–C*H*_2_), 4.89 (dd, 1H, *J*_1_ = 8.7 Hz, *J*_2_ = 3.5 Hz, C*H*(OH)–CH_2_), 7.05 (t, 2H, *J*_1,2_ = 8.6 Hz, Ar–*H*), 7.32–7.37 (m, 2H, Ar–*H*); ^13^C NMR (150 MHz, CDCl_3_): 40.23 (CH(OH)–*C*H_2_), 73.33 (*C*H(OH)–CH_2_), 115.76 (d, *J* = 21.6 Hz, 4-F-*C*_6_H_4_), 127.88 (d, *J* = 8.2 Hz, 4-F-*C*_6_H_4_), 136.25 (d, *J* = 3.1 Hz, 4-F-*C*_6_H_4_), 162.86 (d, *J* = 247.0 Hz, 4-F-*C*_6_H_4_). Anal. Calcd. for C_8_H_8_BrFO (*M*_r_ = 219.05): C, 43.86; H, 3.68. Found: C, 43.63; H 4.01.

#### 4.3.7 (1*R*)-2-bromo-1-(*t*-butyldimethylsilyloxy)-1-(4-fluorophenyl)ethane (8a) and (1*S*)-2-bromo-1-(*t*-butyldimethylsilyloxy)-1-(4-fluorophenyl)ethane (**8b**)

Reaction was carried out in dry conditions under argon atmosphere. For the synthesis of **8a** a solution of imidazole (254 mg, 3.74 mmol) in DMF (0.5 mL) was added to a solution of **7a** (327 mg, 1.49 mmol) in DMF (1 mL) and stirred for 10 min, followed by dropwise addition of TBDMSCl (293 mg, 1.94 mmol) solution in DMF (1.3 mL). The reaction proceeded for 72 h at room temperature. Solvent was evaporated to dryness, distilled water (15 mL) added and the resulting mixture extracted with ethyl acetate (3 × 30 mL). Collected organic layers were dried over Na_2_SO_4_ and solvent evaporated to dryness. **8a** was purified by a silica gel column chromatography (hexane/ethyl acetate 6:1) and obtained as colourless oil (452 mg, 91%). [α]_D_^20^ = −46 (*c* = 1, EtOAc). **8b** was synthesized following the same procedure starting from **7b** (424 mg, 1.94 mmol) and obtained as colourless oil (602 mg, 94%). [α]_D_^20^ = +46 (*c* = 1, EtOAc). FT-IR (KBr) cm^−1^: 2957, 2930, 2887, 2858, 1606, 1509, 1463, 1417, 1362, 1257, 1226, 1155, 1113, 1012, 914, 834, 778, 724, 646. ^1^H NMR (300 MHz, CDCl_3_): −0.09 (s, 3H, Si–C*H*_3_), 0.10 (s, 3H, Si–C*H*_3_), 0.88 (s, 9H, C-(C*H*_3_)_3_), 3.36–3.48 (m, 2H, CH(OTBDMS)-C*H*_2_), 4.82 (dd, 1H, *J*_1_ = 7.4 Hz, *J*_2_ = 4.8 Hz, C*H*(OTBDMS)-CH_2_), 7.02 (t, 2H, *J*_1,2_ = 8.7 Hz, Ar–*H*), 7.28–7.33 (m, 2H, Ar–*H*); ^13^C NMR (75 MHz, CDCl_3_): −4.75 (Si–*C*H_3_), −4.60 (Si–*C*H_3_), 18.35 (*C*-(CH_3_)_3_), 25.86 (C-(*C*H_3_)_3_), 39.48 (CH(OTBDMS)-*C*H_2_), 74.76 (*C*H(OTBDMS)-CH_2_), 115.39 (d, *J* = 21.6 Hz, 4-F-*C*_6_H_4_), 127.98 (d, *J* = 8.1 Hz, 4-F-*C*_6_H_4_), 138.20 (d, *J* = 3.2 Hz, 4-F-*C*_6_H_4_), 162.59 (d, *J* = 245.8 Hz, 4-F-*C*_6_H_4_). Anal. Calcd. for C_14_H_22_BrFOSi (*M*_r_ = 333.31): C, 50.45; H, 6.65. Found: C, 50.17; H 7.05.

#### 4.3.8 (1*R*)-2-iodo-1-(*t*-butyldimethylsilyloxy)-1-(4-fluorophenyl)ethane (9a) and (1*S*)-2-iodo-1-(*t*-butyldimethylsilyloxy)-1-(4-fluorophenyl)ethane (**9b**)

Reaction was carried out in a reaction flask protected from light. To a solution of **8a** (113 mg, 0.34 mmol) in acetone (3 mL), NaI (254 mg, 1.68 mmol) was added. The reaction proceeded at 55 °C for 4 days after which distilled water (15 mL) was added and product extracted with ethyl acetate (3 × 30 mL). Collected organic layers were dried over Na_2_SO_4_ and solvent evaporated to dryness. Thus obtained crude product was purified by a silicagel column chromatography (hexane). Compound **9a** was obtained as a mixture of **9a** and unreacted **8a** (93:7) as light brown oil (124 mg, 96%). **9b** was synthesized following the same procedure starting from **8b** (75 mg, 0.22 mmol) and NaI (168 mg, 1.12 mmol) to give after a silicagel column chromatography (hexane) a mixture of **9b** and unreacted **8b** (94:6) as light brown oil (39 mg, 46%). FT-IR (KBr) cm^−1^: 3448, 2956, 2930, 2887, 2858, 1605, 1509, 1463, 1408, 1362, 1257, 1224, 1106, 997, 887, 837, 776. ^1^H NMR (300 MHz, CDCl_3_): −0.13 (s, 3H, Si–C*H*_3_), 0.10 (s, 3H, Si–C*H*_3_), 0.89 (s, 9H, C-(C*H*_3_)_3_), 3.28–3.30 (m, 2H, CH(OTBDMS)-C*H*_2_), 4.73 (t, 1H, *J*_1,2_ = 6.0 Hz, C*H*(OTBDMS)-CH_2_), 7.00 (t, 2H, *J*_1,2_ = 8.7 Hz, Ar–*H*), 7.26–7.31 (m, 2H, Ar–*H*); ^13^C NMR (75 MHz, CDCl_3_): −4.68 (Si–*C*H_3_), −4.56 (Si–*C*H_3_), 15.03 (CH(OTBDMS)-*C*H_2_), 18.36 (*C*-(CH_3_)_3_), 25.93 (C-(*C*H_3_)_3_), 74.65 (*C*H(OTBDMS)-CH_2_), 115.38 (d, *J* = 21.5 Hz, 4-F-*C*_6_H_4_), 127.80 (d, *J* = 8.2 Hz, 4-F-*C*_6_H_4_), 138.87 (d, *J* = 3.1 Hz, 4-F-*C*_6_H_4_), 162.51 (d, *J* = 246.0 Hz, 4-F-*C*_6_H_4_). HRMS for C_14_H_22_FIOSi (*M*_r_ = 380.31225): calcd. *m/z* [M-H^+^] 379.0396, found 379.0399.

#### 4.3.9 (3*R*,4*R*)-3-[(2*R*)-2-(4-fluorophenyl)-2-(*t*-butyldimethylsilyloxy)ethylamino]-1-(4-fluorophenyl)-4-(4-(*t*-butyldimethylsilyloxy)phenyl)azetidin-2-one (**10a**)

Reaction was carried out in a reaction flask protected from light. To a solution of **2** (154 mg, 0.40 mmol) in CH_3_CN (3 mL) **9a** (151 mg, 0.40 mmol) was added. The reaction proceeded at 80 °C for 7 days after which the solvent was evaporated to dryness. Compound **10a** was purified by a silica gel column chromatography (hexane/ethyl acetate 6:1) and obtained as light brown oil (52 mg, 20%). [α]_D_^20^ = −9 (*c* = 1, EtOAc). FT-IR (KBr) cm^−1^: 3480, 2931, 2858, 1748, 1607, 1505, 1464, 1385, 1259, 1226, 1101, 1006, 914, 834, 515. ^1^H NMR (300 MHz, CDCl_3_): −0.15 (s, 3H, Si–C*H*_3_), 0.02 (s, 3H, Si–C*H*_3_), 0.18 (s, 6H, Si-(C*H*_3_)_2_), 0.86 (s, 9H, C-(C*H*_3_)_3_), 0.96 (s, 9H, C-(C*H*_3_)_3_), 2.01 (bs, 1H, CH(OTBDMS)–CH_2_–N*H*), 2.78–2.99 (m, 2H, CH(OTBDMS)-C*H*_2_-NH), 4.00 (d, 1H, *J* = 2.1 Hz, C4 β-lactam), 4.61 (d, 1H, *J* = 1.8 Hz, C3 β-lactam), 4.77 (dd, 1H, *J*_1_ = 7.8 Hz, *J*_2_ = 4.3 Hz, C*H*(OTBDMS)–CH_2_–NH), 6.81 (d, 2H, *J* = 8.7 Hz, Ar–*H*), 6.91 (t, 2H, *J*_1,2_ = 8.8 Hz, Ar–*H*), 6.99 (t, 2H, *J*_1,2_ = 8.8 Hz, Ar–*H*), 7.13 (d, 2H, *J* = 8.5 Hz, Ar–*H*), 7.21–7.30 (m, 4H, Ar–*H*); ^13^C NMR (75 MHz, CDCl_3_): −4.75 (Si–*C*H_3_), −4.41 (Si–*C*H_3_), −4.28 (Si-(*C*H_3_)_2_), 18.29 (*C*-(CH_3_)_3_), 25.73 (C-(*C*H_3_)_3_), 25.96 (C-(*C*H_3_)_3_), 55.69 (CH(OTBDMS)-*C*H_2_-NH), 63.86 (C4 β-lactam), 74,18 (*C*H(OTBDMS)–CH_2_–NH), 75.73 (C3 β-lactam), 115.24 (d, *J* = 21.3 Hz, 4-F-*C*_6_H_4_), 115.90 (d, *J* = 22.6 Hz, 4-F-*C*_6_H_4_), 118.92 (d, *J* = 7.9 Hz, 4-F-*C*_6_H_4_), 120.84 (4-OTBDMS-*C*_6_H_4_), 127.23 (4-OTBDMS-*C*_6_H_4_), 127.95 (d, *J* = 8.0 Hz, 4-F-*C*_6_H_4_), 129.40 (4-OTBDMS-*C*_6_H_4_), 133.78 (d, *J* = 2.7 Hz, 4-F-*C*_6_H_4_), 138.86 (d, *J* = 3.0 Hz, 4-F-*C*_6_H_4_), 156.11 (4-OTBDMS-*C*_6_H_4_), 159.20 (d, *J* = 243.6 Hz, 4-F-*C*_6_H_4_), 162.33 (d, *J* = 245.5 Hz, 4-F-*C*_6_H_4_), 166.38 (C=O, β-lactam). HRMS for C_35_H_48_F_2_N_2_O_3_Si_2_ (*M*_r_ = 638.93503): calcd. *m/z* [M+Na^+^] 661.3063, found 661.3082.

#### 4.3.10 (3*R*,4*R*)-3-[(2*S*)-2-(4-fluorophenyl)-2-(*t*-butyldimethylsilyloxy)ethylamino]-1-(4-fluorophenyl)-4-(4-(*t*-butyldimethylsilyloxy)phenyl)azetidin-2-one (**10b**)

**10b** was synthesized following the procedure for **10a** and obtained as light brown oil (9 mg, 19%). [α]_D_^20^ = +16 (*c* = 1, EtOAc). FT-IR (KBr) cm^−1^: 3439, 2929, 2857, 1749, 1608, 1506, 1472, 1385, 1259, 1101, 1012, 913, 833, 516. ^1^H NMR (300 MHz, CDCl_3_): −0.14 (s, 3H, Si–C*H*_3_), 0.04 (s, 3H, Si–C*H*_3_), 0.18 (s, 6H, Si-(C*H*_3_)_2_), 0.86 (s, 9H, C-(C*H*_3_)_3_), 0.96 (s, 9H, C-(C*H*_3_)_3_), 1.80 (bs, 1H, CH(OTBDMS)–CH_2_–N*H*), 2.80–2.96 (m, 2H, CH(OTBDMS)-C*H*_2_-NH), 4.02 (d, 1H, *J* = 2.0 Hz, C4 β-lactam), 4.62 (d, 1H, *J* = 2.0 Hz, C3 β-lactam), 4.81 (dd, 1H, *J*_1_ = 7.6 Hz, *J*_2_ = 4.3 Hz, C*H*(OTBDMS)–CH_2_–NH), 6.81 (d, 2H, *J* = 8.6 Hz, Ar–*H*), 6.90 (t, 2H, *J*_1,2_ = 8.8 Hz, Ar–*H*), 6.99 (t, 2H, *J*_1,2_ = 8.7 Hz, Ar–*H*), 7.12 (d, 2H, *J* = 8.5 Hz, Ar–*H*), 7.20–7.31 (m, 4H, Ar–*H*); ^13^C NMR (75 MHz, CDCl_3_): −4.72 (Si–*C*H_3_), −4.39 (Si–*C*H_3_), −4.27 (Si-(*C*H_3_)_2_), 18.30 (*C*-(CH_3_)_3_), 25.74 (C-(*C*H_3_)_3_), 25.97 (C-(*C*H_3_)_3_), 55.68 (CH(OTBDMS)-*C*H_2_-NH), 63.35 (C4 β-lactam), 74,25 (*C*H(OTBDMS)–CH_2_–NH), 75.82 (C3 β-lactam), 115.25 (d, *J* = 21.5 Hz, 4-F-*C*_6_H_4_), 115.91 (d, *J* = 22.8 Hz, 4-F-*C*_6_H_4_), 118.94 (d, *J* = 7.9 Hz, 4-F-*C*_6_H_4_), 120.84 (4-OTBDMS-*C*_6_H_4_), 127.25 (4-OTBDMS-*C*_6_H_4_), 127.96 (d, *J* = 8.0 Hz, 4-F-*C*_6_H_4_), 129.35 (4-OTBDMS-*C*_6_H_4_), 133.77 (d, *J* = 2.8 Hz, 4-F-*C*_6_H_4_), 138.89 (d, *J* = 3.0 Hz, 4-F-*C*_6_H_4_), 156.13 (4-OTBDMS-*C*_6_H_4_), 159.22 (d, *J* = 243.0 Hz, 4-F-*C*_6_H_4_), 162.34 (d, *J* = 245.4 Hz, 4-F-*C*_6_H_4_), 166.36 (C=O, β-lactam). HRMS for C_35_H_48_F_2_N_2_O_3_Si_2_ (*M*_r_ = 638.93503): calcd. *m/z* [M+H^+^] 639.3244, found 639.3256.

#### 4.3.11 (3*R*,4*R*)-3-[(2*R*)-2-(4-fluorophenyl)-2-hydroxyethylamino)]-1-(4-fluorophenyl)-4-(4-hydroxyphenyl)azetidin-2-one (**5**)

Compound **10a** (24 mg, 0.04 mmol) was dissolved in 3% HCl in ethanol (1.59 mL) and the mixture was stirred at room temperature overnight. Solvent was evaporated to dryness and crude product purified by a silica gel column chromatography (hexane/ethyl acetate 1:3, 5% MeOH). Product **5** was obtained as light yellow powder (13 mg, 84%). mp 136–138 °C. [α]_D_^20^ = −18 (*c* = 1, EtOAc). FT-IR (KBr) cm^−1^: 3448, 1735, 1617, 1509, 1389, 1225, 1102, 833. ^1^H NMR (300 MHz, DMSO-*d*_*6*_): 2.75–2.77 (m, 2H, CH(OH)–C*H*_2_–NH), 2.95 (bs, 1H, CH(OH)–CH_2_–N*H*), 3.97 (bs, 1H, C4 β-lactam) 4.58–4.63 (m, 1H, C*H*(OH)–CH_2_–NH), 4.84 (d, 1H, *J* = 2.0 Hz, C3 β-lactam), 5.33 (d, 1H, *J* = 4.4 Hz, CH(O*H*)–CH_2_–NH), 6.74 (d, 2H, *J* = 8.5 Hz, Ar–*H*), 7.08–7.24 (m, 8H, Ar–*H*), 7.33–7.37 (m, 2H, Ar–*H*), 9.46 (s, 1H, Ar–O*H*); ^13^C NMR (75 MHz, DMSO-*d*_*6*_): 54.55 (CH(OH)–*C*H_2_–NH), 62.34 (C4 β-lactam), 71.45 (*C*H(OH)–CH_2_–NH), 75.47 (C3 β-lactam), 114.60 (d, *J* = 21.0 Hz, 4-F-*C*_6_H_4_), 115.66 (4-OH-*C*_6_H_4_), 115.80 (d, *J* = 21.7 Hz, 4-F-*C*_6_H_4_), 118.65 (d, *J* = 8.0 Hz, 4-F-*C*_6_H_4_), 127.49 (4-OH-*C*_6_H_4_), 127.52 (4-OH-*C*_6_H_4_), 127.84 (d, *J* = 8.1 Hz, 4-F-*C*_6_H_4_), 133.87 (d, *J* = 2.3 Hz, 4-F-*C*_6_H_4_), 140.40 (d, *J* = 2.7 Hz, 4-F-*C*_6_H_4_), 157.31 (4-OH-*C*_6_H_4_), 158.08 (d, *J* = 240.7 Hz, 4-F-*C*_6_H_4_), 161.17 (d, *J* = 241.6 Hz, 4-F-*C*_6_H_4_), 167.10 (C=O, β-lactam). HRMS for C_23_H_20_F_2_N_2_O_3_ (*M*_r_ = 410.41331): calcd. *m/z* [M+H^+^] 411.1515, found 411.1517.

### 4.4 Spectrophotometric titration

Stock solution of amino alcohols **5** and **6** (160 μL, 2 mg/mL methanol) was added to NaCl solution in water (20 mL, 0.1 mol/L), thus resulting in a solution of each amino alcohol **5** and **6** in concentration of 4 × 10^−5^ mol/L. Furthermore, solutions of NaOH (0.1 mol/L, 1 mol/L and 2 mol/L) were prepared in NaCl solution in water (100 mL, 0.1 mol/L). NaOH solutions (1.5–20 μL) were added to each solution of amino alcohol **5** or **6**, pH values measured and UV spectra (*λ* 200–350 nm) recorded at pH 6–12. The titrations were performed at rt (20 °C) and inflection points of sigmoid curves at *λ* 247 nm were calculated as p*K*_a_ values for both amino alcohols.

### 4.5 Cell culture and establishment of the cell line stably expressing hNPC1L1 protein

Madin–Darby Canine Kidney II (MDCKII) cells, MDCKII cells stably expressing human NPC1L1 (hNPC1L1/MDCKII), and human HepG2 liver cells were maintained in DMEM (Dulbecco's Modified Eagle Medium, Invitrogen, USA) containing 100 units/mL penicillin and 100 μg/mL streptomycin supplemented with 10% FCS (Sigma–Aldrich, Germany). The cells were grown at 37 °C in a humidified 5% CO_2_ incubator. Stable transfection of MDCKII cells with human NPC1L1 protein was performed using Lipofectamine LTX (Invitrogen, USA) according to the manufacturer's protocol. Selection with 1 mg/mL G418 (Sigma–Aldrich, Germany) was started 2 days after transfection. The concentration of G418 was decreased to 500 μg/mL after 14 days. Stably transfected cells were maintained in DMEM containing 100 units/mL penicillin and 100 μg/mL streptomycin supplemented with 10% FCS and 500 μg/mL G418.

### 4.6 Cytotoxicity assay

MDCKII, hNPC1L1/MDCKII, and HepG2 cells were seeded in 96-well microtiter plates on day 0 at densities of 3000 cells/well. On day 1, tested compounds (73 mM stock solution in DMSO) were added in five consecutive 10-fold dilutions (10^−8^ to 10^−4^ mol/L) and incubated for 72 h. Cytotoxicity of the compounds was assessed on day 4 by the MTT cell proliferation assay (Sigma–Aldrich, Germany) which detects mitochondrial dehydrogenase activity in viable cells [33]. The results are expressed as LC_50_ and the values for each compound were calculated from dose–response curves using linear regression analysis. Each concentration was tested in quadruplicate in three independent experiments. Toxicity of the compounds in combination with micelles was also tested using the same method. MDCKII and hNPC1L1/MDCKII were seeded in 96-well microtiter plates (150 μL medium/well) at a density of 2 × 10^5^ cells/mL medium 24 h before the experiment. Micelles were prepared according to the modified method reported by Field et al. [34]. In brief, 0.25 mM oleic acid, 50 μM cholesterol, 10 μM compactin, 50 μM mevalonate, 5 mM Na-taurocholate (Sigma–Aldrich, Germany) in DMEM/10% FCS were mixed and sonicated. Following the micellar formation, compounds **1**, **5**, **6**, and **5**/**6** (70:30) (73 mM stock solution in DMSO) were added to the medium and vortexed to obtain the final concentrations of the compounds (25, 50, 100, 150, 200 μM) and applied to the cells for 1 h. Thereafter, cytotoxicity was determined using the MTT assay.

### 4.7 Medium preparation

Medium A contained DMEM plus 100 units/mL penicillin and 100 μg/mL streptomycin supplemented with 5% lipoprotein-deficient serum (density > 1.21 g/mL, prepared from FCS by ultracentrifugation). Cholesterol Replenishing Medium (Medium B) was prepared according to the modified method reported by Field et al. [34]. In brief, 0.25 mM oleic acid, 50 μM free cholesterol, 10 μM compactin, 50 μM mevalonate, 5 mM Na-taurocholate (Sigma–Aldrich, Germany), and [^3^H]cholesterol (0.18 μCi/mL medium, [1,2–3H(N)] cholesterol, 1 mCi/mL (ARC Inc., USA)) in Medium A were mixed and sonicated. Following the micellar formation, compounds **1**, **5**, **6**, **5**/**6** (70:30), and **6**/**5** (85:15) (73 mM stock solution in DMSO) were added to Medium B and vortexed to obtain the final concentrations (10, 30, 60, 90, 120, 150, 200 μM).

### 4.8 *In vitro* cholesterol uptake assay

MDCKII wildtype and hNPC1L1/MDCKII cells were seeded in 24-well plates at a density of 5 × 10^5^ cells/mL medium (500 μL medium/well) in Medium A 24 h before the treatment. Cells were washed once with PBS and incubated in Medium B (500 μL medium/well) containing micelles and tested compounds in indicated concentrations for 1 h. Following the incubation, the medium was removed and cells were washed three times with ice-cold 0.2% free fatty acid-free BSA. Cells were lysed and radioactivity in the lysate (100 μL) was determined by liquid scintillation counting. Protein concentrations of the lysates were determined with the DC Protein Assay (Bio-Rad, USA). The results were calculated as cpm/mg protein and expressed as percentage of inhibition compared to untreated cells. Each concentration was tested in duplicate in three independent experiments.

### 4.9 *In vivo* acute cholesterol absorption

Male C57BL/6 mice aged 25–28 weeks were maintained under a 12 h light/12 h dark cycle in a temperature-controlled environment with free access to chow diet (Ssniff, Germany) and water. For 2 days, mice (*n* = 3–5) were fasted for 4 h and gavaged with corn oil (100 μl) (vehicle) containing ezetimibe **1** (10 mg/kg/day) or compounds **5**, **6**, and **5**/**6** (70:30) (10 or 20 mg/kg/day). On day 2, 90 min post vehicle or compound treatment, mice were gavaged with corn oil (200 μl) containing [^3^H]cholesterol (2 μCi) ([1,2–3H(N)] cholesterol, 1 mCi/mL, ARC Inc., St. Louis, USA). Plasma, liver, and small intestines were collected 4 h post-gavage. Cholesterol uptake and absorption were determined as previously described [35].

### 4.10 Statistical analyses

The results are presented as mean ± S.D. or mean ± S.E.M. Statistical analyses were performed using unpaired two-tailed *t*-test or one-way analysis of variance (ANOVA) followed by Dunnett's test for multiple comparisons using GraphPad Prism 5.0 software (San Diego, CA, USA). Differences were considered significant at *P* < 0.05. IC_50_ values were determined with GraphPad Prism 5.0.
