# Supplementary material for: Novel amino-β-lactam derivatives as potent cholesterol absorption inhibitors
Source: Eur J Med Chem. 2014 Nov 24;87:722–34. doi: 10.1016/j.ejmech.2014.10.014 (PMC4237514; doi:10.1016/j.ejmech.2014.10.014)
Supplement: Supplementary file 1 [file mmc1.doc]

**Supporting information**

**Novel Amino-β-lactam Derivatives as Potent Cholesterol Absorption Inhibitors**

Tonko Dražić ^a^,*, Krešimir Molčanov ^a^, Vinay Sachdev ^b^, Martina Malnar ^a^, Silva Hećimović ^a^, Jay V. Patankar ^b,1^, Sascha Obrowsky ^b^, Sanja Levak-Frank ^b^, Ivan Habuš ^a^, and Dagmar Kratky ^b^,*

^a^ *Ruđer Bošković Institute, Bijenička c. 54, HR-10002 Zagreb, Croatia,* ^b^ *Institute of Molecular Biology and Biochemistry, Medical University of Graz, Graz, Austria, ^1^ present address: Centre for Molecular Medicine and Therapeutics, Department of Medical Genetics, University of British Columbia, Vancouver, Canada*

Table of contents:

^1^H and ^13^C NMR spectra of compounds **2**, **4**, **7a and b**, **8a and b**, **9a and b**, **10a and b**, **5**, **6**

Western blotting

Western blot analysis of hNPC1L1 expression: Figure S1

Cytotoxicity of compounds **5**, **6,** and **5**/**6** (70:30) in Medium B in MDCKII wildtype and hNPC1L1/MDCKII cells: Figure S2

Inhibition of cholesterol uptake in hNPC1L1 MDCKII cells with ezetimibe **1**: Figure S3

^1^H NMR spectrum of **2**

^13^C NMR spectrum of **2**

^1^H NMR spectrum of **4**

^13^C NMR spectrum of **4**

^1^H NMR spectrum of **7a** and **7b**

^13^C NMR spectrum of **7a** and **7b**

^1^H NMR spectrum of **8a** and **8b**

^13^C NMR spectrum of **8a** and **8b**

^1^H NMR spectrum of **9a** and **9b**

^13^C NMR spectrum of **9a** and **9b**

^1^H NMR spectrum of **10a**

^13^C NMR spectrum of **10a**

^1^H NMR spectrum of **10b**

^13^C NMR spectrum of **10b**

^1^H NMR spectrum of **5**

^13^C NMR spectrum of **5**

^1^H NMR spectrum of **6**

^13^C NMR spectrum of **6**

**Western blotting.** Cells were washed with PBS and lysed in Co-IP buffer (50 mM Tris pH 7.6, 150 mM NaCl, 2 mM EDTA, 1% NP-40) supplemented with protease inhibitor cocktail (Roche Diagnostics GmbH, Germany). Total protein concentrations were measured using DC Protein Assay (Bio-Rad, USA). For immunoblotting analysis, aliquots of cell lysates were mixed with 6x sample buffer (60% glycerol, 12% SDS, 3% DTT, 1/8 v/v 0.5 M Tris pH 6.8, bromophenol blue), loaded (55 μg protein) on an 8% SDS–PAGE gel, and then transferred to a PVDF membrane (Roche Diagnostics GmbH, Germany). The blot was incubated with anti-NPC1L1 antibody (1:500; Novus Biologicals, USA) followed by incubation with HRP-conjugated anti-rabbit secondary antibody (1:5000; Bio-Rad, USA). Equal protein loading was confirmed using anti-actin (1:1000; Sigma Aldrich, Germany) primary antibody. Proteins were visualized by chemiluminescence using BM Chemiluminescence Western Blotting Substrate (Roche Diagnostics GmbH, Germany) on documentation system from UVItec Cambridge.


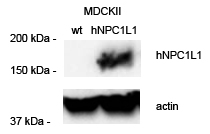


**wt hNPC1L1**

**hNPC1L1**

**β-actin**

**200 kDa -**

**150 kDa -**

**37 kDa -**

**MDCKII**

**Figure S1.** Western blot analysis of hNPC1L1 expression. MDCKII wildtype (wt) and hNPC1L1/MDCKII cell lysates were analyzed by Western blotting to determine the expression of human NPC1L1 in MDCKII (control) and MDCKII cells stably transfected with human NPC1L1 using an anti-NPC1L1 antibody. The expression of β-actin was determined as loading control.

**
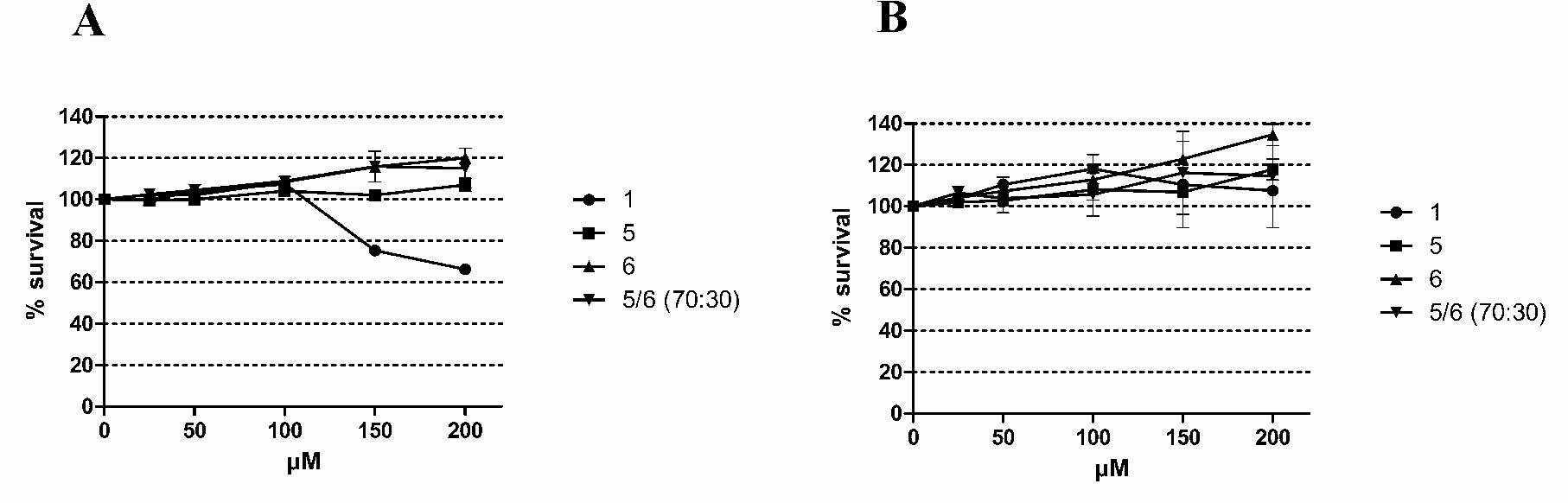
**

**Figure S2.** *In vitro* cytotoxicity of the compounds **5**, **6,** and **5**/**6** (70:30) in combination with micelles. Cytotoxicity of the newly synthesized compounds was determined in (**A**) MDCKII wildtype and (**B**) hNPC1L1/MDCKII cells. The cells were incubated with the indicated concentrations of the compounds in Medium B (0.25 mM oleic acid, 50 μM free cholesterol, 10 μM compactin, 50 μM mevalonate, 5 mM Na-taurocholate in DMEM supplemented with 5% LPDS) for 1 h. The results are expressed as percentage of the survival of cells treated with the compound compared to untreated cells. Data represent mean ± S.E.M. of three independent experiments.


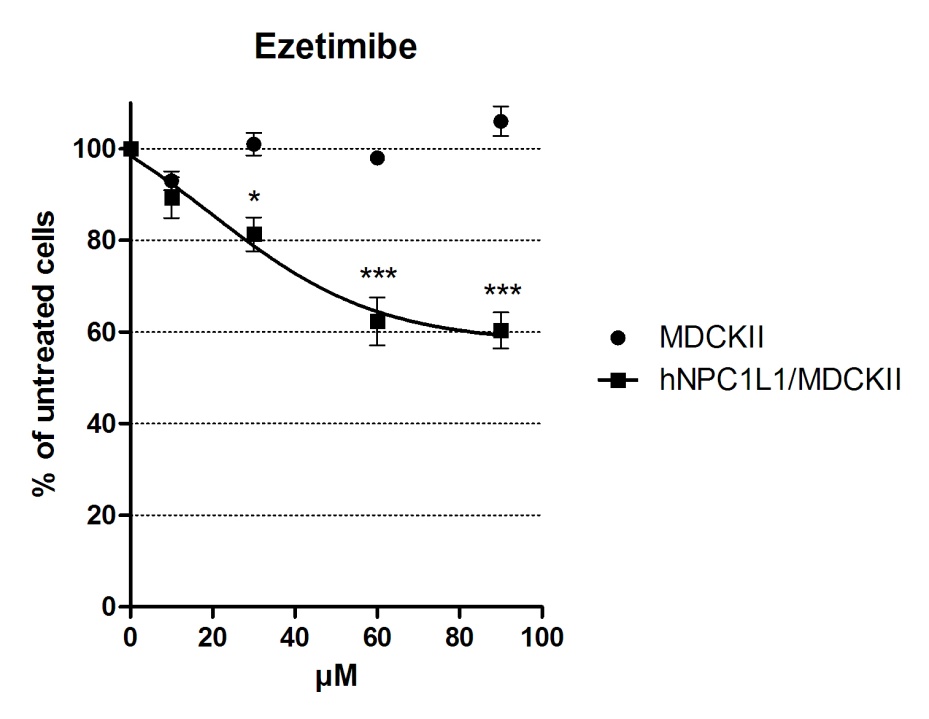


**Figure S3.** Ezetimibe **1** inhibits cholesterol uptake in hNPC1L1/MDCKII cells. Cholesterol uptake was determined in MDCKII wildtype and hNPC1L1/MDCKII cells. Cells were treated with the indicated concentrations of ezetimibe **1** for 1 h and cholesterol uptake was determined as described under „Experimental section“. The results are expressed as percentage of inhibition compared to untreated cells. Data represent mean ± S.E.M. of three independent experiments. **p* < 0.05, *** *p* < 0.001 determined by one-way ANOVA followed by Dunnett's test.
